# Gastrointestinal *Talaromyces marneffei* infection in a patient with AIDS: A case report and systematic review

**DOI:** 10.3389/fimmu.2022.980242

**Published:** 2022-09-30

**Authors:** Fangmei Ling, Tao Guo, Junrong Li, Yidong Chen, Mingyang Xu, Shuang Li, Liangru Zhu

**Affiliations:** ^1^ Division of Gastroenterology, Union Hospital, Tongji Medical College, Huazhong University of Science and Technology, Wuhan, China; ^2^ Department of Pathology, Union Hospital, Tongji Medical College, Huazhong University of Science and Technology, Wuhan, China

**Keywords:** *Talaromyces marneffei*, talaromycosis, intestine, HIV, endoscopy

## Abstract

*Talaromyces marneffei* is a thermally dimorphic fungus that affects multiple organs and frequently invades immunocompromised individuals. However, only a few studies have reported the presence of intestinal infection associated with *T. marneffei*. Herein, we reported a case of intestinal *T. marneffei* infection in a man who complained of a 1-month history of intermittent fever, abdominal pain, and diarrhea. The result of the human immunodeficiency virus antibody test was positive. Periodic acid-Schiff and Gomorrah’s methylamine silver staining of the intestinal biopsy tissue revealed *T. marneffei* infection. Fortunately, the patient’s symptoms rapidly resolved with prompt antifungal treatment. In addition, we summarized and described the clinical characteristics, management, and outcomes of patients with intestinal *T. marneffei* infection. A total of 29 patients were identified, the majority of whom (65.52%) were comorbid with acquired immunodeficiency syndrome. The main clinical features included anemia, fever, abdominal pain, diarrhea, weight loss, and lymphadenopathy. The transverse and descending colon, ileocecum, and ascending colon were the most common sites of lesions. A considerable number of patients (31.03%) developed intestinal obstruction, intestinal perforation, and gastrointestinal bleeding. Of the 29 patients, six underwent surgery, 23 survived successfully with antifungal treatment, five died of *T. marneffei* infection, and one died of unknown causes. *T. marneffei* intestinal infection should be considered when immunodeficient patients in endemic areas present with non-specific symptoms, such as fever, abdominal pain, and diarrhea. Appropriate and timely endoscopy avoids delays in diagnosis. Early aggressive antifungal therapy improves the clinical outcomes of patients.

## Introduction

*Talaromyces marneffei*, an emerging pathogenic thermally dimorphic fungus that causes mycosis with multiple organ involvement in humans, was formerly known as *Penicillium marneffei* (1). Despite a period of nearly half a century since it was first reported in 1973, *T. marneffei* has not received significant attention from researchers (2). Tropical regions, such as southern China, India, Thailand, Vietnam, and Southeast Asia, are susceptible to *T. marneffei* infection (1) (3). In general, *T. marneffei* develops in immunocompromised hosts, especially in patients infected with human immunodeficiency virus (HIV), and can be hematogenously disseminated to multiple systems, such as the respiratory, skin, and digestive systems (3). As an opportunistic pathogenic fungus, it rarely invades individuals without apparent risk factors or under immunocompromised conditions. In addition, intestinal involvement of *T. marneffei* is rarely reported, accounting for 1.9% of all *T. marneffei* infections (4). Mononuclear phagocytes are the main system that is usually invaded by *T. marneffei* and are abundant in the mucosa of the digestive tract. In addition to reviews and retrospective studies, 28 cases of intestinal *T. marneffei* with well-defined histopathological features have been reported in detail, most of whom are comorbid with acquired immune deficiency syndrome (AIDS). Here, we reported a case of *T. marneffei* infection involving the intestine of a patient with AIDS and reviewed the clinical characteristics and endoscopic findings to improve the ability to identify the disease.

## Case presentation

**Figure 1:**
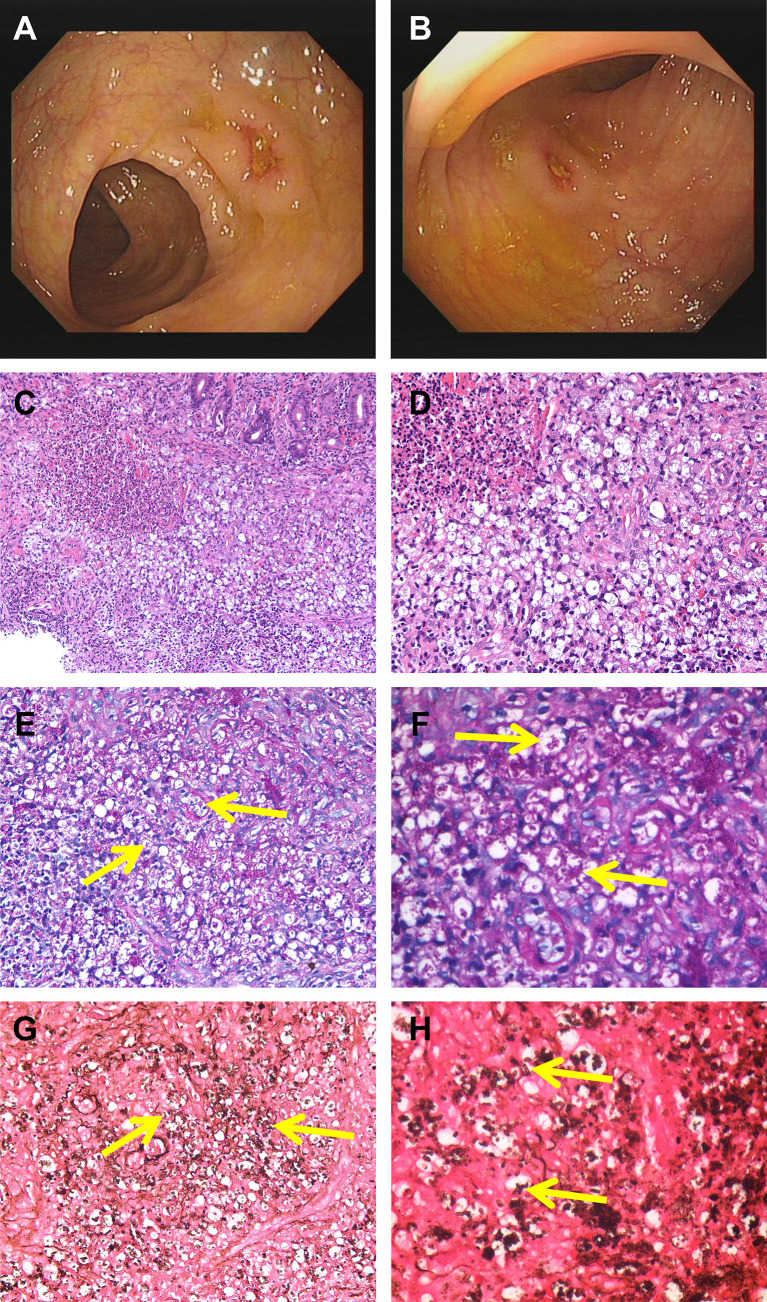
Colonoscopy revealed scattered ulcers with unknown etiology located at transverse colon **(A)** and the ascending colon **(B)**. H&E staining showed infiltration of clustered microorganisms and granulomas in the lamina propria at ×200 **(C)** and ×400 **(D)**. PAS staining revealed some intracellular and extracellular yeasts (yellow arrow) distributed in the colon at ×400 **(E)** and higher magnification **(F)**. GMS staining showed abundantly septate yeast-like microorganisms (yellow arrow) at ×400 **(G)** and higher magnification **(H)**. PAS, periodic acid-Schiff; GMS, Gomorrah’s methylamine silver.

A 53-year-old man from Hubei Province, China, was admitted to our hospital with a 1-month history of intermittent fever (highest temperature, >39°C), abdominal pain, diarrhea, fatigue, and weight loss associated with anorexia and cough. However, the patient had lived in Guangdong Province for a long period of time. Half a month before the patient’s admission, an upper gastrointestinal (GI) endoscopy revealed chronic non-atrophic gastritis with erosion, fungal esophagitis, and xanthoma of the gastric corpus, without specific intervention. During the patient’s hospital visit, a physical examination revealed the following: temperature, 37.8°C; pulse rate, 107/min; respiratory rate, 20/min; and blood pressure, 117/85 mmHg. The rest of the patient’s physical examination results were unremarkable. Laboratory examinations showed low levels of hemoglobin (98 g/L), leukocytes (3.17 × 10^9^/L), albumin (26.8 g/L), and prealbumin (0.062 g/L), increased levels of high-sensitivity C-reactive protein (78.44 mg/L), erythrocyte sedimentation rate (73 mm/h), neutrophils (76.1%), D-dimer (1.55 mg/L), and antinuclear antibody (1:100) and a positive result for the cytomegalovirus (CMV)-DNA (<4.00E+02 copies/ml). The patient’s absolute neutrophil count was in the normal range (2.41 × 10^9^/L). Electrolyte parameters were abnormal, with hyponatremia (129.0 mmol/L) and hypochloremia (88.8 mmol/L). Anti-HIV antibody was positive, and the patient’s CD4 T count was 10 cells/μl, accounting for 2.16% of total T lymphocytes. The rest of the blood tests, including immunoglobulin, complement 3 (C3) and 4 (C4), hepatic transaminase, creatinine, extractable nuclear antigen, antineutrophil cytoplasmic antibody, and tuberculosis T-SPOT, were within the normal range. Alpha-fetoprotein, carcinoembryonic antigen, severe acute respiratory syndrome coronavirus 2 nucleic acid, and blood culture results were negative. Chest computed tomography (CT) revealed a thickened and blurred lung texture and multiple enlarged lymph nodes (abdominal, retroperitoneal, bilateral axillary, and supraclavicular). Abdominal ultrasound was non-contributory, except for gallbladder polypoid. A colonoscopy revealed multiple scattered and swollen ulcers in the ileocecal region and ascending, transverse, descending, and sigmoid colon, and multiple site biopsies were performed (**Figures 1A, B**). Ulcers were characterized by clear boundaries, with moss attached to the bottom of the erosions, and the largest ulcer (approximately 1.0 × 1.5 cm) was located in the transverse colon. The demarcation between the normal and flared areas was clear. Therefore, the patient was treated with cefixime, mesalazine, and ilaprazole for 1 week before the completion of colonic histopathology, but without clinical improvement. After 7 days, histopathological evaluation of the biopsies using hematoxylin and eosin staining revealed a chronic ulcer of the colonic mucosa with fungal granuloma and focal small abscess (**Figures 1C, D**). Next, biopsy tissues were specifically stained with periodic acid-Schiff (PAS) and Gomorrah’s methylamine silver (GMS) stains. Briefly, the former involves staining with periodate followed by binding with Schiff’s solution to reveal glycogen and polysaccharides in the tissue, whereas the latter involves the oxidation of periodic acid followed by the reaction of the aldehyde group with hexamine silver to expose metallic silver. PAS staining (**Figures 1E, F**) and GMS (**Figures 1G, H**) staining of the intestinal biopsy specimens were positive. Fungal spores with a transverse septum were observed in the tissue cells, which were presented in the form of short fusiform or sausage-shaped spores aggregated into mulberry shapes. Combined with the results of immunohistochemistry, cluster of differentiation (CD) 68(+), pan-cytokeratin(−), CD34(−), S100(−), and CMV(−) confirmed *T. marneffei* infection. Subsequently, the patient received antifungal therapy with intravenous fluconazole (400 mg once daily) and intravenous ganciclovir (250 mg twice daily) for 5 days, followed by oral fluconazole (200 mg once daily) for 9 days and ganciclovir (1,000 mg once daily) for 23 days. During hospitalization, the patient was continuously administered symptomatic treatment, such as mesalazine, amino acid, and alanyl-glutamine. A change in the antifungal treatment with fluconazole led to defervescence, and the patient’s symptoms improved quickly. The patient was discharged from our hospital and maintained oral antifungal agents.

## Literature search and results

### Procedures of systematic review

**Figure 2:**
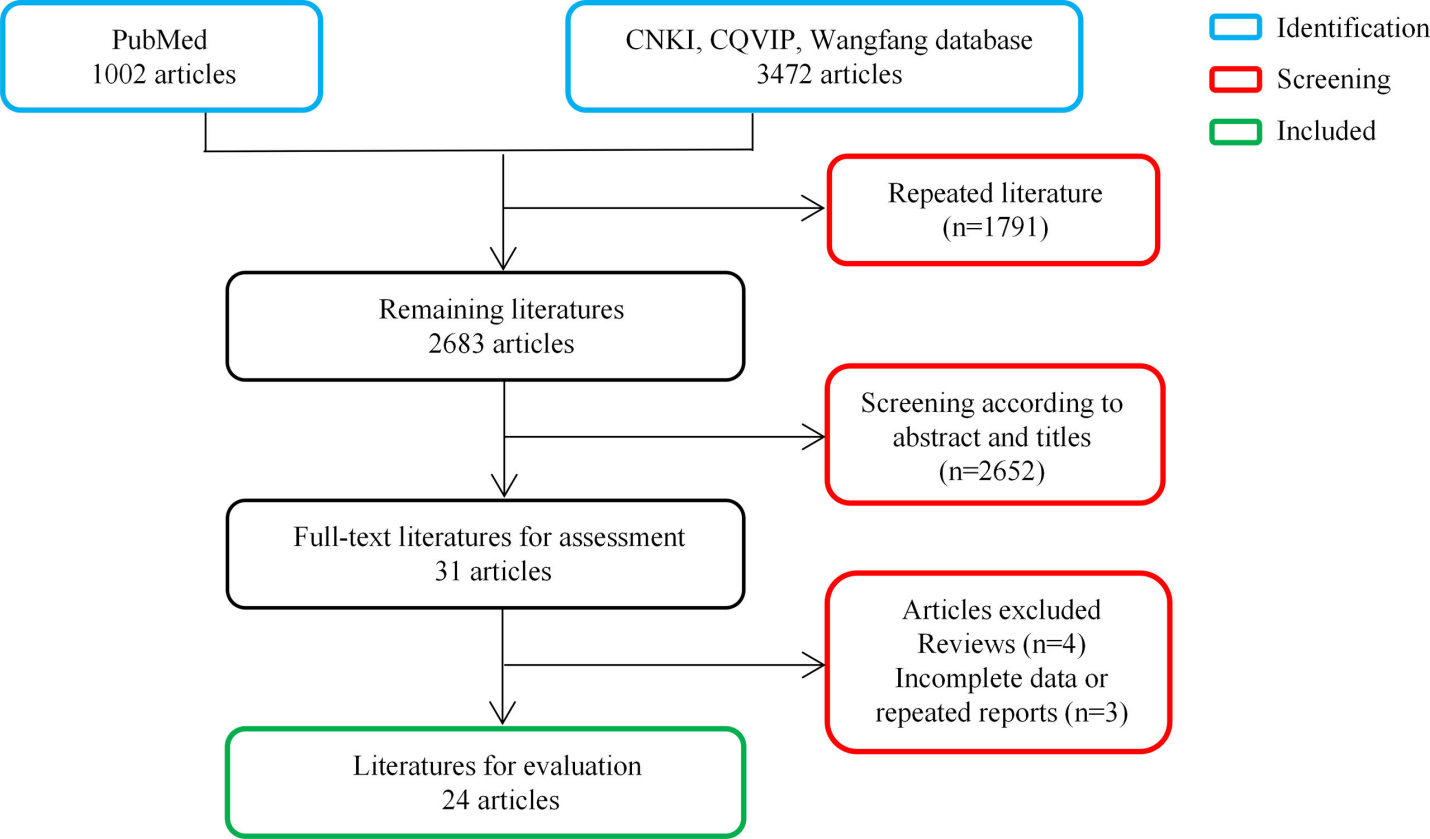
Flow diagram for selection of articles.

PubMed, CNKI, CQVIP, and Wanfang electronic databases in both Chinese and English were searched from inception to 31 April 2022, using the following term combinations: *Talaromyces marneffei*, *Penicillium marneffei*, talaromycosis, and penicilliosis. All published literature on *T. marneffei* infection associated with intestinal involvement was reviewed to investigate its characteristics. After the screening of the titles, abstracts, and full texts, 24 studies with 28 patients were included in this review. A flowchart of the study selection procedure is presented in **Figure 2**.

### Inclusion and exclusion criteria

The cases were retrieved using the following inclusion criteria 1) diagnosis of local or disseminated *T. marneffei* infection based on international consensus (5) 2) *T. marneffei* identified by direct microscopy, culture, histopathology, and sequencing of specimens obtained from autopsy, surgery, or endoscopic biopsy 3) an affected site involving the GI system and clinical syndrome consistent with intestinal infection; and 4) available data with detailed information. We excluded the following 1) reviews or duplicate studies and 2) cases with abdominal symptoms with no evidence of GI involvement.

### Data extraction

Information from each eligible patient, including reported year and region, age, sex, course of the disease, main presenting symptoms, laboratory findings, diagnostic methods, antifungal treatment, and outcomes, was collected for data extraction and tabulated.

### Results

**Figure 3:**
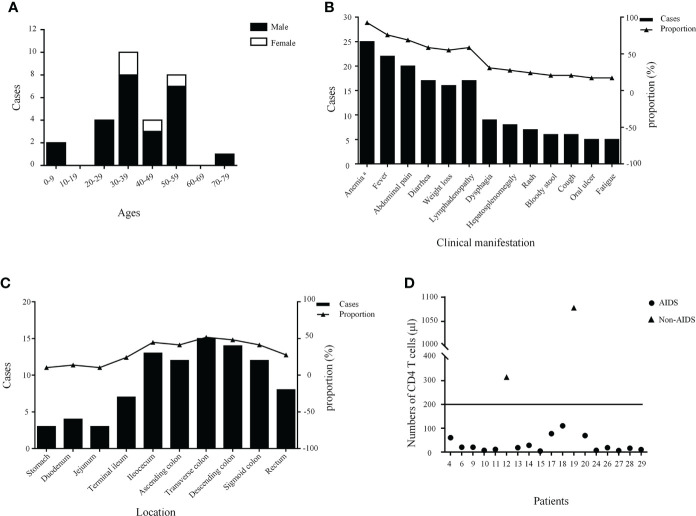
The age stratification and gender distribution **(A)**, clinical manifestations **(B)**, lesion distribution **(C)**, and the number of CD4 T cells of patients **(D)**. ^a^ A total of 27 cases had hemoglobin values.

In total, 17 English and seven Chinese articles with 28 patients were identified according to a literature search (4, 6–28). Overall, the number of reported cases remains single digit despite increased exposure in recent years. The detailed characteristics of the 29 patients with intestinal *T. marneffei* infection are presented in **Table 1**. There were 25 male and four female patients, most of whom were reported in mainland China. Their median age was 38 (range, 0.33–72) years. The age group and gender distribution of patients are shown in **Figure 3A**, with middle-aged and young adults being the most common. Of the 29 patients, 24 with underlying diseases among immunocompromised hosts, comprising 19 HIV-infected patients, accounted for 65.52%. The common clinical manifestations and distribution of colonic infections are presented in **Figure 3B**. Most patients had constitutional symptoms, such as anemia (92.59%), fever (75.86%), abdominal pain (68.97%), diarrhea (58.62%), weight loss (55.17%), and lymphadenopathy (58.62%). As shown in **Figure 3C**, *T. marneffei* infections involved the transverse colon (51.72%), descending colon (48.28%), ileocecum (44.83%), ascending colon (41.38%), and sigmoid colon (41.38%). A small percentage of patients (13.79%) had lesions located in the upper GI tract. Ulcers and erosions (92.59%) were dominant among those who underwent endoscopy described in the literature. CD4 cell counts were provided in 18 patients, of whom 16 had a count of <200 cells/µl (**Figure 3D**). Lymphadenopathy (58.33%) and bowel wall thickening (50.00%) were the common phenomena provided by abdominal CT. Fungi were detected in the blood, marrow, skin, lymph nodes, stomach, and omentum majus, except for the intestine. Nearly one-third of the patients (9/29, 31.03%) developed bowel-related complications, manifested as bowel obstruction (10.34%), bowel perforation (3.45%), and lower GI bleeding (13.79%). Of these, six patients underwent surgery owing to severe GI symptoms. Most patients (23/29, 79.31%) survived with antifungal treatment. The detailed clinical characteristics of the 29 patients with GI *T. marneffei* infection are presented in **Supplementary Table 1**.

**Table 1:** Clinical Characteristics Concerning 29 Patients With Gastrointestinal Talaromycosis.

Characteristics	Patients (n = 29)
Area of report	
China	27 (27/29, 93.10%)
India	2 (2/29, 6.90%)
Age at diagnosis (years) (median, IQR)	38 (32, 50.25)
Male	25 (25/29, 86.21%)
Medical History	25 (25/29. 86.21%)
AIDS	19 (19/29, 65.52%)
TB	3 (3/29, 10.34%)
Syphilis	2 (2/29, 6.90%)
Renal transplant	1 (1/29, 3.45%)
SLE	1 (1/29, 3.45%)
Waldenstrom macroglobulinemia, ITP, PBC	1 (1/29, 3.45%)
Autoimmune haemolytic anaemia	1 (1/29, 3.45%)
STAT3 mutation	1 (1/29, 3.45%)
HBV carriers	1 (1/29, 3.45%)
Disease course (months) (median, IQR)^a^	1.5 (0.5, 3)
Anemia^a^	25 (25/27, 92.59%)
Intestinal complications	9 (9/29,31.03%)
Obstruction	3 (3/29, 10.34%)
Perforation	1 (1/29, 3.45%)
Obstruction & Perforation	1 (1/29, 3.45%)
Gastrointestinal bleeding	4 (4/29, 13.79%)
Site(s) of positive culture/histology	
Intestine	27 (27/29, 93.10%)
Omentum majus	1 (1/29, 3.45%)
Blood	9 (9/29, 31.03%)
Skin	4 (4/29, 13.79%)
Marrow	6 (6/29, 20.69%)
llymph nodes	4 (4/29, 13.79%)
Endoscopy^a^	
Erosion /ulceration	24 (24/27, 88.89%)
Hemorrhage	4 (4/27, 14.81%)
Abdomen CT^b^	
Lymphadenopathy	7 (7/12, 58.33% )
Bowel-wall thickening	6 (6/12, 50.00% )
Intestinal obstruction	3 (3/12, 25.00% )
Hepatomegaly/Splenomegaly	3 (3/12, 25.00% )
Edematous intestine	2 (2/12, 16.67% )
Treatment	
Antifungal treatment	23 (23/29, 79.31%)
Surgery	6(6/29, 20.69%)
None	2(2/29, 6.90%)
Outcome	
Survival	23 (23/29, 79.31%)
Died	5 (5/29, 17.24%)
Died of unknown reasons	1 (1/29, 3.45%)

AIDS, acquired immunodeficiency syndrome; CT, computed tomography; ITP, idiopathic thrombocytopenic purpura; PBC, primary biliary cirrhosis; PR, present report; TB, tuberculosis.

^a^27 caces provided haemoglobin values, endoscopic findings and disease course; ^b^12 caces provided abdomen CT descriptions.

## Discussion

From 1988 to 2022, in addition to the review, only 28 other cases of *T. marneffei* infection identified *via* abdominal symptoms, intestinal lesions, and histopathology involving the digestive tract were observed in the existing literature, with the detailed information provided. According to the report, GI symptoms account for approximately 18.8%–31% of *T. marneffei* infection, but the proportion does not match the morbidity of actual confirmed GI infections with *T. marneffei* (4, 11, 20, 28). For example, in a retrospective analysis, only three of 33 *T. marneffei* infections with GI symptoms were diagnosed as *T. marneffei* GI system infections (20). Although the cases of penicilliosis in this study did not represent the true number of cases, the estimates of the disease characteristics of intestinal penicilliosis were provided. It is speculated that the lack of reports or data linking *T. marneffei* to GI symptoms is attributed to the accessibility of specimen acquisition. Compared to easily accessible specimens, such as the skin, blood, and lymph nodes, anatomical sites of the GI tract are more difficult to access. However, the diagnosis usually requires an endoscopically guided histopathological examination of the intestinal tract. The presence of skin or superficial lymph node lesions in an individual may result in more rapid initiation of empirical treatment, the GI symptoms will resolve, and the need for endoscopy will be reduced at this time. Therefore, *T. marneffei* infection involving the digestive tract may be considered rare, especially in patients diagnosed by digestive endoscopy and tissue biopsy. In a few previously published papers, a review of 19 patients from 1988 to 2020 summarized the clinical characteristics of the cases (20). The demonstration of the details was different from that of our study, as the table displays the clinical characteristics one by one. A large number of intestinal *T. marneffei* infection case reviews have been published recently, including 31 cases in a monocentric retrospective study over the past 10 years (29). None of these studies discussed the occurrence and incidence of intestinal complications and surgery. Apart from the above studies, a few reports containing a large number of patients with talaromycosis involving the digestive tract have been published. Here, we present a novel case of *T. marneffei* intestinal involvement and systematically review the literature to determine its clinical course, diagnosis, and management.

Immunocompromised individuals are susceptible to *T. marneffei* infection of the intestine, and most cases have been reported among patients with AIDS in areas where the organism is endemic (3). However, in recent years, it has been increasingly diagnosed in individuals with other immunosuppressive diseases, undergoing solid organ transplantations, with malignancies, receiving novel anti-cancer-targeted therapies, and with autoimmune and primary immunodeficiency diseases (30). Here, the majority of patients diagnosed with *T. marneffei* infection with intestinal involvement were immunocompromised individuals, especially individuals with AIDS (65.52%), followed by tuberculosis (10.34%). Coincidentally, patients with CD4 counts <200 cells/μl were perfectly matched with those with AIDS. A low CD4 cell count (<200 cells/μl) is defined by the World Health Organization (WHO) as an advanced HIV disease, indicating poor immunity and a high risk for *T. marneffei* infection (3). The routine laboratory tests in the patient in our report were not specific, except for anti-HIV antibodies with a CD4 T-cell count <200 cells/μl. The incidence and severity of *T. marneffei* infection among people living with HIV and AIDS have changed since the beginning of the widespread era of antiretroviral therapy (ART). Based on the results of a systematic review of 33 eligible studies, the prevalence of *T. marneffei* infection was 5.3% in the limited ART era and decreased to 2.5% in the widespread ART era (31). However, no statistically significant differences were observed between the groups. Notably, a portion of the study reported that prehospital ART slightly reduced the risk of mortality in patients with *T. marneffei* infection from 1.92 to 1.58, with a reduction in disease severity and clinical manifestations (32, 33). As the access to ART and early diagnosis of HIV increase in the future, the prevalence of *T. marneffei* infection is likely to decline. However, the distant goal of 90% of people living with HIV on ART declared by the WHO and the large HIV population suggest that several people remain at risk for *T. marneffei* infection.

GI *T. marneffei* infection usually presents with subacute symptoms characterized by fever, abdominal pain, diarrhea, weight loss, and lymphadenopathy, consistent with previous reports (4, 20, 29). Recently, according to Ying et al., the prevalence of anemia in HIV-associated talaromycosis was 95.6% (34). Here, we reported a similar proportion of anemia at 92.6% in patients with intestinal *T. marneffei* infection. *T. marneffei* attacks any segment of the digestive tract, notably the colon. The cecum and transverse, ascending, and descending colon are commonly affected by ulcers and erosions, which are relatively rare in the small intestine, stomach, and esophagus. In the present case, prominent manifestations on endoscopy were unrepresentative and had limited contribution to the diagnosis. Abdominal CT, which reflects the signs of tissue and organ involvement, such as hepatosplenomegaly, lymphadenitis, intestinal wall thickening, and luminal narrowing, is helpful for diagnosis. Positive culture, molecular techniques (polymerase chain reaction and next-generation sequencing), and histopathology are indispensable for accurate diagnosis, as disseminated *T. marneffei* infection usually affects the blood, skin, bone marrow, lymph nodes, lungs, liver, and spleen (35). Therefore, considering the high diagnostic yield for multiple organisms and the risk of intestinal biopsy, *T. marneffei* identification *via* histopathology of intestinal tissue has rarely been described (20). It is plausible that an individual who experiences serious intestinal complications or a difficult clinical situation free from biopsy might lead to an underdiagnosis. In our systematic literature review, a considerable number of patients were diagnosed with *T. marneffei* infection in the reticuloendothelial system, such as the skin, bone marrow, blood, and lymph nodes. For a definitive diagnosis of *T. marneffei* infection based on fungal culture, the sensitivities of the bone marrow, skin, and blood were 100%, 90%, and 76%, respectively (36). Unfortunately, whether the patient in the present case report had disseminated *T. marneffei* infection remains unclear because of negative blood cultures and the absence of biopsies from other sites. Clinically, timely endoscopy under comprehensive consideration identifies the affected sites and severity of disease for early diagnosis and targeted treatment to prevent serious complications, such as bowel perforation and intestinal obstruction.

In the present case, the clinical characteristics, laboratory examination, imaging, and endoscopic manifestations were almost compatible with those summarized in previous reviews. Fever, diarrhea, and abdominal pain, the most common complaints, were the initial symptoms of the patient in this case report (4, 20, 29). Given similar predisposing factors and overlapping clinical features (anemia, fever, and extensive lymphadenopathy), the differential diagnosis of GI tumors, lymphoma, Crohn’s disease, and intestinal tuberculosis should be considered. Immediately thereafter, an endoscopy with pathological biopsy was performed, and multiple diffuse ulcers were observed. Conventional therapy with thymosin and mesalazine provided little benefit within 10 days of waiting for histopathology. Fortunately, the baseline conditions, immune function, and organ function of the patient closely related to clinical outcomes were relatively good. A timely and coherent medical procedure enabled the patient to obtain successful treatment without complications.

Complications of intestinal obstruction, intestinal perforation, and GI bleeding occurred in 31.0% of patients in our study, of which 55.6% received surgical intervention. Xinling et al. reported a case of death due to GI bleeding and hemorrhagic shock (10). A retrospective analysis comprising 27 cases of deaths in patients with AIDS with *T. marneffei* infection suggested a 40.7% incidence rate of GI bleeding (37). The incidence of complications in patients with intestinal *T. marneffei* infection has not been discussed and determined because of the small number of cases. Therefore, the establishment of a systematic predictive model requires rigorous, multicenter, large-sample, prospective studies. In the current study, among the five cases of death due to *T. marneffei* infection, two were complicated by severe intestinal complications (GI bleeding). The destruction of pathogens and immunodeficiencies in patients may be a possible explanation for the high mortality rate. In particular, diagnostic and treatment modalities remain inadequate and delayed as therapy often begins 1 or 2 weeks after microbiological results are provided. The G and GM tests may quickly indicate deep fungal infections.

The mortality rate is up to 24%–33% after antifungal therapy and even higher in untreated patients with disseminated *T. marneffei* infection (3, 35, 38). The mortality rate will exponentially increase to 50% when the diagnosis is delayed and significantly up to 100% when the diagnosis is missed (3). Despite the high morbidity rate of this tropical infectious disease, the factors predicting disease progression and clinical outcomes remain poorly understood. The mortality rate is higher in non-HIV-infected patients with *T. marneffei* infection than in HIV-infected patients, reflecting delayed or misdiagnosed diagnosis due to lack of clinical suspicion (35). Similarly, four patients (4/5, 80%) who died in our review were non-HIV-infected patients, who had a higher mortality rate than HIV-infected patients (36.3% *vs.* 5.6%). All three patients who were untreated with antifungal drugs were declared dead. The prognosis can be improved with active and standard antifungal therapies. Both amphotericin B and itraconazole have been approved for the treatment of *T. marneffei* infection. Amphotericin B resulted in higher clinical remission and fungal clearance and a lower risk of death at week 24 as compared with itraconazole (21% *vs.* 11.6, p = 0.006) (39). The currently recommended regimen for HIV-infected patients is 2 weeks of amphotericin B followed by 10 weeks of itraconazole until CD4 cells are >100/μl for 6 months. However, the duration of this therapeutic schedule is not well-defined in non-HIV-infected patients (30). The patient in our center intravenously received antifungal therapy, and his symptoms significantly improved within 3 days.

This systematic review and report have some limitations. First, the cases we included were all searched in an electronic database in Chinese and English. However, we were unable to retrieve articles published in other languages. We believe that there were more patients infected with GI *T. marneffei* infection that were missed, such that the number of included cases was less than the actual number of infections. Second, the clinical information for all patients was obtained from published articles. The presentations and descriptions provided to the patients varied among the articles, which led to the incompleteness of our information collection, especially in earlier years. Third, an incomplete reporting of population characteristics and an insufficiently large number of cases prevented us from inferring factors that influenced the clinical outcome, which limits our ability to identify patients with high mortality. Fourth, the viral load of patients with HIV in the current study and other reports was not available.

In conclusion, GI *T. marneffei* infection should be considered in immunocompromised individuals who complain of non-specific intestinal symptoms, especially in endemic areas, and individuals previously exposed to HIV. Familiarity with the form of infection would facilitate clinician vigilance and reduce the probability of GI *T. marneffei* infection being misdiagnosed or overlooked. Optimal diagnostic and treatment approaches are urgently required to save the lives of patients in the early stages of the disease.

## Data availability statement

The raw data supporting the conclusions of this article will be made available by the authors, without undue reservation.

## Ethics statement

Written informed consent was obtained from the individual(s) for the publication of any potentially identifiable images or data included in this article.

## Author contributions

FL completed the main body of the manuscript. JL and YC participated in information collection and data analysis. MX and SL were responsible for the documentation. LZ provided the main ideas for writing the final article. All authors contributed to the article and approved the submitted version.

## Funding

This work is supported by the Major Projects of the Ministry of Science and Technology of China (2018YFC0114600) and National Natural Science Foundation of China (81873558 and 82170547).

## Conflict of interest

The authors declare that the research was conducted in the absence of any commercial or financial relationships that could be construed as a potential conflict of interest.

## Publisher’s note

All claims expressed in this article are solely those of the authors and do not necessarily represent those of their affiliated organizations, or those of the publisher, the editors and the reviewers. Any product that may be evaluated in this article, or claim that may be made by its manufacturer, is not guaranteed or endorsed by the publisher.

## References

[1] Supparatpinyo K Khamwan C Baosoung V Nelson KE Sirisanthana T. Disseminated penicillium marneffei infection in southeast Asia. Lancet (1994) 344:110–3. doi: 10.1016/s0140-6736(94)91287-4 7912350

[2] DiSalvo AF Fickling AM Ajello L. Infection caused by penicillium marneffei: description of first natural infection in man. Am J Clin Pathol (1973) 60:259–63. doi: 10.1093/ajcp/60.2.259 4720403

[3] Narayanasamy S Dat VQ Thanh NT Ly VT Chan JF-W Yuen K-Y. A global call for talaromycosis to be recognised as a neglected tropical disease. Lancet Glob Health (2021) 9:e1618–22. doi: 10.1016/S2214-109X(21)00350-8 PMC10014038 34678201

[4] Zhou Y Liu Y Wen Y. Gastrointestinal manifestations of talaromyces marneffei infection in an HIV-infected patient rapidly verified by metagenomic next-generation sequencing: a case report. BMC Infect Dis (2021) 21:376. doi: 10.1186/s12879-021-06063-1 33882850 PMC8059157

[5] Donnelly JP Chen SC Kauffman CA Steinbach WJ Baddley JW Verweij PE. Revision and update of the consensus definitions of invasive fungal disease from the European organization for research and treatment of cancer and the mycoses study group education and research consortium. Clin Infect Dis (2020) 71:1367–76. doi: 10.1093/cid/ciz1008 PMC7486838 31802125

[6] Tsang DN Chan JK Lau YT Lim W Tse CH Chan NK. Penicillium marneffei infection: an underdiagnosed disease? Histopathology (1988) 13:311–8. doi: 10.1111/j.1365-2559.1988.tb02041.x 3056826

[7] Deng Z Ribas JL Gibson DW Connor DH. Infections caused by penicillium marneffei in China and southeast Asia: review of eighteen published cases and report of four more Chinese cases. Rev Infect Dis (1988) 10:640–52. doi: 10.1093/clinids/10.3.640 3293165

[8] Tsui WM Ma KF Tsang DN. Disseminated penicillium marneffei infection in HIV-infected subject. Histopathology (1992) 20:287–93. doi: 10.1111/j.1365-2559.1992.tb00985.x 1577408

[9] Leung R Sung JY Chow J Lai CK. Unusual cause of fever and diarrhea in a patient with AIDS. Penicillium marneffei infection Dig Dis Sci (1996) 41:1212–5. doi: 10.1007/BF02088239 8654154

[10] Xinling B Ying W Jun G. Penicilliosis marneffei manifested by symptoms of gut firstly: a case report. Chin J Integr Med (2004) 18:47–8. doi: 10.3969/j.issn.1001-7089.2004.01.027

[11] Ko CI Hung CC Chen MY Hsueh PR Hsiao CH Wong JM. Endoscopic diagnosis of intestinal penicilliosis marneffei: report of three cases and review of the literature. Gastroint Endosc (1999) 50:111–4. doi: 10.1016/s0016-5107(99)70359-7 10385737

[12] Hongzhou L Lianguo L Yinzhong S. Colonic pathologic changes in an AIDS patient complicated with penicillosis. J Microbes Infect (2006) 1:157–60. doi: 10.3969/j.issn.1673-6184.2006.03.008

[13] George IA Sudarsanam TD Pulimood AB Mathews MS. Acute abdomen: an unusual presentation of disseminated penicillium marneffei infection. Indian J Med Microbiol (2008) 26:180–2. doi: 10.4103/0255-0857.40538 18445960

[14] Hung H-G Lok K-H. Intestinal penicillium marneffei: an unusual cause of chronic diarrhea in an AIDS patient. J Dig Dis (2010) 11:189–91. doi: 10.1111/j.1751-2980.2010.00435.x 20579223

[15] Chan JFW Chan TSY Gill H Lam FYF Trendell-Smith NJ Sridhar S. Disseminated infections with talaromyces marneffei in non-AIDS patients given monoclonal antibodies against CD20 and kinase inhibitors. Emerg Infect Dis (2015) 21:1101–6. doi: 10.3201/eid2107.150138 PMC4816330 26079984

[16] Yanhua L Guihong C Shengnan L. A case of colonic ulcerative AIDS with penicillium marneffei infection under colonoscopy. Chin J Dig Endos (2016) 33:411–2. doi: 10.3760/cma.j.issn.1007-5232.2016.06.019

[17] Shi F Xiaoling W Xiuming Z. Pathological diagnosis of a rare intestinal penicillium marneffei infection in an acquired immunodeficiency syndrome patient: a case report and literature review. Int J Clin Exp Pathol (2017) 10:3710–5.

[18] Lijuan C Xiujiang H Shuiqing L. Acquired immunodeficiency syndrome with penicillium marneffei infection involving the intestine: a case report. Chin J Dig (2017) 37:201–2. doi: 10.3760/cma.j.issn.0254-1432.2017.03.016

[19] Zhao Y-K Liu J-Y Liu J-H Lu S Wu H-H Luo D-Q. Recurrent talaromyces marneffei infection presenting with intestinal obstruction in a patient with systemic lupus erythematosus. Mycopathologia (2020) 185:717–26. doi: 10.1007/s11046-020-00469-2 32647906

[20] Pan M Huang J Qiu Y Zeng W Li Z Tang S. Assessment of talaromyces marneffei infection of the intestine in three patients and a systematic review of case reports. Open Forum Infect Dis (2020) 7:ofaa128. doi: 10.1093/ofid/ofaa128 32523970 PMC7264840

[21] Philip R Sridhar Coelho VV Roopavathana B Chase S. Opportunistic penicilliosis infection causing intestinal obstruction in people living with HIV complicating antiretroviral therapy. BMJ Case Rep (2020) 13:e230121. doi: 10.1136/bcr-2019-230121 PMC7046417 32060105

[22] Pan Y Tong JL Ran ZH Cao ZJ. Talaromyces (Penicillium) infection in a patient presenting with intestinal ulcers mimicking inflammatory bowel disease. J Dig Dis (2020) 21:301–3. doi: 10.1111/1751-2980.12872 32378793

[23] Yu J Lu Y Zhuang Y Shi Q. A case of drug-related non-HIV infection of talaromyces marneffei. Chin J Inflamm Bowel Dis (2020) 04:349–50. doi: 10.3760/cma.j.cn101480-20200330-00039

[24] Yang Y Liang X Huang S. Disseminated talaromyces marneffei infection mimicking intestinal tuberculosis. Lancet Infect Dis (2021) 21:1469. doi: 10.1016/S1473-3099(21)00269-3 34562404

[25] Fu F. A case of intestinal infection with penicillium marneffei and literature review. Healthmust-Readmagazine (2020) 15:212.

[26] He L Huang Y Chen X Wu Y Wang X Jian S. A case of multiple intestinal ulcers in an HIV-infected patient caused by talaromyces marneffei. Chin J AIDS STD (2021) 27:313–4. doi: 10.13419/j.cnki.aids.2021.03.27

[27] Tan Y Zhang Z Wu M Zou S Guo W Liang K. Unusual disseminated talaromyces marneffei infection presenting with fever and diarrhea in an AIDS patient: a case report and literature review. Acta Clin Belg (2022), 1–4. doi: 10.1080/17843286.2022.2067957 35467497

[28] Cui X Su F Ye H Jiang Y Guo X. Disseminated talaromycosis complicated by recurrent gastrointestinal bleeding and hemorrhagic shock: a case report. BMC Infect Dis (2022) 22:238. doi: 10.1186/s12879-022-07230-8 35264100 PMC8905750

[29] Xie Z Lai J Peng R Mou M Liang H Ning C. Clinical characteristics of HIV-associated talaromyces marneffei infection of intestine in southern China. Int J Infect Dis (2022) 120:48–50. doi: 10.1016/j.ijid.2022.03.057 35398298

[30] Cao C Xi L Chaturvedi V. Talaromycosis (Penicilliosis) due to talaromyces (Penicillium) marneffei: Insights into the clinical trends of a major fungal disease 60 years after the discovery of the pathogen. Mycopathologia (2019) 184:709–20. doi: 10.1007/s11046-019-00410-2 31811603

[31] Qin Y Huang X Chen H Liu X Li Y Hou J. Burden of talaromyces marneffei infection in people living with HIV/AIDS in Asia during ART era: a systematic review and meta-analysis. BMC Infect Dis (2020) 20:551. doi: 10.1186/s12879-020-05260-8 32727383 PMC7392840

[32] Jiang J Meng S Huang S Ruan Y Lu X Li JZ. Effects of talaromyces marneffei infection on mortality of HIV/AIDS patients in southern China: a retrospective cohort study. Clin Microbiol Infect (2019) 25:233–41. doi: 10.1016/j.cmi.2018.04.018 29698815

[33] Shi X Yan Q Zhan Y Shi C Song F Wang L. Effect of combination antiretroviral therapy on the clinical manifestations, radiological characteristics, and disease severity of HIV-associated talaromyces marneffei infection. Int J STD AIDS (2020) 31:747–52. doi: 10.1177/0956462420925248 32631212

[34] Ying RS Le T Cai WP Li YR Luo CB Cao Y. Clinical epidemiology and outcome of HIV-associated talaromycosis in guangdong, China, during 2011-2017. HIV Med (2020) 21:729–38. doi: 10.1111/hiv.13024 PMC7978497 33369035

[35] Chan JFW Lau SKP Yuen K-Y Woo PCY. Talaromyces (Penicillium) marneffei infection in non-HIV-infected patients. Emerg Microbes Infect (2016) 5:e19. doi: 10.1038/emi.2016.18 26956447 PMC4820671

[36] Vanittanakom N Cooper CR Fisher MC Sirisanthana T. Penicillium marneffei infection and recent advances in the epidemiology and molecular biology aspects. Clin Microbiol Rev (2006) 19:95–110. doi: 10.1128/CMR.19.1.95-110.2006 16418525 PMC1360277

[37] Zhang JS Hong WX Li LH Tang XP Cheng XJ. Retrospectively analyze 27 cases of death in AIDS patients with Penicilliosis marneffei. J Pract Med (2014), 30 13:2108–10. doi: 10.3969/j.issn.1006-5725.2014.13.031

[38] Klus J Ly VT Chan C Le T. Prognosis and treatment effects of HIV-associated talaromycosis in a real-world patient cohort. Med Mycol (2021) 59:392–9. doi: 10.1093/mmy/myab005 PMC8023982 33644813

[39] Le T Kinh NV Cuc NTK Tung NLN Lam NT Thuy PTT. A trial of itraconazole or amphotericin b for HIV-associated talaromycosis. N Engl J Med (2017) 376:2329–40. doi: 10.1056/NEJMoa1613306 28614691

