# Supplementary material for: Gastrointestinal Talaromyces marneffei infection in a patient with AIDS: A case report and systematic review
Source: Front Immunol. 2022 Sep 30;13:980242. doi: 10.3389/fimmu.2022.980242 (PMC9561240; doi:10.3389/fimmu.2022.980242)
Supplement: Supplementary file 1 [file Table_1.docx]

Supplementary Table 1. Clinical characteristics concerning of 29 Patients co-infected with gastrointestinal Talaromyces Marneffei

| Patient | Year | Area | Sex | Age  (years) | Medical History | Clinical manifestation | Enlargement of lymph node | CD4+ (cells/ ul) | Endoscopy manifestation | Location | Intestinal complications | Organs involved | Management | Outcome |
| --- | --- | --- | --- | --- | --- | --- | --- | --- | --- | --- | --- | --- | --- | --- |
| 1 | 1988 | Hong Kong, China | M | 58 | Autoimmune haemolytic anaemia | Fever, abdominal pain, hepatosplenomegaly, anemia | None | Not mentioned | Not mentioned | Descending and sigmoid colon | None | Liver, colon, lung  Biopsy, autopsy, culture | Amphotericin B; surgery | Died |
| 2 | 1988 | Guangxi, China | M | 0.33 | None | Anemia, hepatosplenomegaly, fever, and diarrhea | Not mentioned | Not mentioned | Not mentioned | Not mentioned | Not mentioned | Liver, spleen,  bowel, mesenteric lymph node, bone marrow, kid-  ney, lung, adrenal, and cerebral meninges  Autopsy, histopathology | None | Died |
| 3 | 1992 | Hong Kong, China | M | 72 | AIDS | Anorexia; Dysphagia; lose weight; Gastrointestinal hemorrhage | Not mentioned | Not done | Ulcer and bleeding | Jejunum | Gastrointestinal bleeding | Small intestine, mesenteric lymph node, liver  Biopsy, autopsy, culture | Surgery | Died |
| 4 | 1996 | Hong Kong, China | M | 32 | AIDS, TB | Fever, anemia, diarrhea, cough and hepatomegaly | None | 60 | Multiple solitary ulcers | Ileocecum, transverse and descending colon | None | Colon  Biopsy, culture | Amphotericin B and itraconazole. | Survived |
| 5 | 1999 | Taiwan, China | M | 33 | Renal transplantation, TB | Anemia, cough, bloody stool, oral ulcer, anemia | None | Not mentioned | Erosion and bleeding | Stomach, duodenum | None | Duodenum, blood  Biopsy, culture | None | Died |
| 6 | 1999 | Taiwan, China | M | 52 | AIDS | Fever, diarrhea, abdominal pain, rash, anemia, hepatomegaly | None | 20 | Shallow ulcers | Ileocecum | None | Ieoileocecum, blood, bone marrow and skin  Biopsy, culture | Amphotericin B, itraconazole | Survived |
| 7 | 1999 | Taiwan, China | M | 30 | AIDS | Dyspepsia, abdominal pain, diarrhea fever, bloody stool, weight loss, anemia, | Mesenteric | Not mentioned | Multiple solitary shallow ulcers | Ileocecum, ascending and transverse colons | None | Colon, blood  Biopsy, culture | Amphotericin B, itraconazole | Survived |
| 8 | 2004 | Shanghai, China | M | 21 | TB | Abdominal pain, abdominal distension, anorexia, rash, fever, bloody stool, anemia, fatigue | Neck | Not mentioned | Not mentioned | Terminal ileum | Gastrointestinal bleeding | Colon, skin  Biopsy, culture, histopathology | Itraconazole | Died |
| 9 | 2006 | Shanghai, China | M | 51 | AIDS | Diarrhea, fever, rash, weight loss, anorexia, anemia, fatigue, hepatosplenomegaly | Intra-abdominal, supraclavicular, elbow | 20 | Multiple scattered shallow ulcers | Transverse colon, descending colon and sigmoid colon | None | Colon, blood, skin, lymph nodes  Biopsy, culture | Fluconazole, itraconazole | Survived |
| 10 | 2008 | Indian | M | 33 | AIDS | Fever, abdominal pain, weight loss, anemia， anorexia, vomit | Axillary, mesenteric | 7 | Nodular erythematous | Duodenum | Intestinal obstruction | Duodenum, marrow  Biopsy, culture | Amphotericin B, itraconazole | Survived |
| 11 | 2010 | Hong Kong, China | M | 39 | AIDS | Diarrhea, weight loss, fever, anemia, sore throat, oral ulcer | None | 11 | Multiple small aphthous ulcers | Entire colon, stomach, duodenum | None | Colon, stomach, duodenum  Biopsy, culture | Amphotericin B, itraconazole | Survived |
| 12 | 2015 | Hong Kong, China | M | 56 | Waldenström macroglobulinemia, ITP, PBC | Fever, night sweating, cough, bloody diarrhea | None | 315 | Multiple shallow ulcers | Terminal ileum | None | Feces, terminal ileal and nasopharyngeal  Biopsy, culture | Amphotericin B, voriconazole | Survived |
| 13 | 2016 | Guangxi, China | M | 41 | AIDS | Abdominal pain, abdominal distension, fever, cough, weight loss, oral ulcer, anemia, | Intra-abdominal | 18 | Multiple ulcers | Sigmoid colon, descending colon | None | Colon  Biopsy | Amphotericin B, itraconazole | Survived |
| 14 | 2017 | Zhejiang, China | M | 52 | AIDS | Fever, abdominal pain, diarrhea, weight loss, anorexia, anemia | Mesenteric | 28 | Multiple solitary and shallow ulcers | Transverse colon, descending colon, sigmoid  colon and rectum | None | Colon  Biopsy, histopathology | Itraconazole | Survived |
| 15 | 2017 | Guizhou, China | F | 32 | AIDS, HBV carriers | Abdominal pain, diarrhea, abdominal distension, fever, jaundice, night sweat, oral ulcer, hepatosplenomegaly, weight loss, ulcerationofvulva, anemia | Neck, intra-abdominal, retroperitoneal | 4 | Erosion | Terminal ileum, rectum | None | Colon, blood  Biopsy, culture | Amphotericin B, itraconazole | Survived |
| 16 | 2020 | Guangdong, China | F | 51 | SLE | Abdominal pain, fever, nausea, abdominal distension, constipation, anemia, dysphagia | None | Not mentioned | Enteritis of ileitis | Jejunum, terminal ileum, | Intestinal obstruction | Small intestine  Histopathology | Itraconazole; surgery | Died of unknown reasons |
| 17 | 2020 | Guangxi, China | M | 37 | AIDS | Abdominal pain, diarrhea, weight loss, fever, hepatomegaly, anemia | Neck and inguinal | 77 | Scattered annular ulcer and circular protuberance erosion | Ileocecum, entire colon | None | Colon, lung, liver, , blood, and lymph node  Biopsy, culture | Amphotericin B, itraconazole | Survived |
| 18 | 2020 | Guangxi, China | M | 50 | AIDS | Abdominal pain, anemia, weight loss | None | 110 | Ulcers | Ileocecum, entire colon | None | Colon  Biopsy | Amphotericin B, fluconazol，itraconazole | Survived |
| 19 | 2020 | Guangxi, China | M | 3.08 | STAT3 mutation | Abdominal pain, fever, hepatomegaly, anemia | Neck region, mesenteric | 1078 | Erosion, ulcers, polypoid lesions | Ileocecum, entire colon | Intestinal obstruction, intestinal perforation | Colon, liver, marrow, lymph node  Biopsy, culture | Voriconazole  surgery | Survived |
| 20 | 2020 | India | F | 38 | AIDS | Abdominal pain, vomit, weight loss, rash, anorexia, anemia | Mediastinal | 69 | Ulcers, luminal narrowing | Jejunum | Intestinal obstruction | Jejunum, skin  Biopsy | Amphotericin B, itraconazole, surgery | Survived |
| 21 | 2020 | Shanghai, China | M | 27 | None | Abdominal pain, diarrhea, weight loss, bloody stool, anemia | Neck | Not mentioned | Multiple erosions and ulcers | Terminal ileum, Ileocecum, ascending colon, transverse colon | Intestinal perforation | Ileum, sputum, lymph node  Biopsy, culture | Amphotericin B, itraconazole, surgery | Survived |
| 22 | 2020 | Guangdong, China | M | 20 | None | Fever, bloody stool, weight loss, rash | Intra-abdominal, retroperitoneal | Not mentioned | Erosion, ulcers | Terminal ileum, ileocecum, entire colon | None | Colon  Biopsy | Itraconazole, | Survived |
| 23 | 2021 | Guangdong, China | M | 27 | None | Fever, coughing, diarrhoea, abdominal pain, and weight loss | None | Not mentioned | Multiple annular ulcers | Ileocecum | None | ileocecum, blood, sputum  Biopsy, culture | Amphotericin B, voriconazole | Survived |
| 24 | 2021 | Guangdong, China | M | 33 | AIDS | Fever, abdominal pain, night sweats, anorexia, fatigue, weight loss, diarrhea,anemia | Intra-abdominal, etroperitoneal | 7 | Multiple small shallow ulcers, bleeding | Ileocecum, ascending colon, transverse colon and descending colon | Gastrointestinal bleeding | Omentum majus, colon  Biopsy, metagenomic next-generation sequencing (mNGS) | Amphotericin B, itraconazole | Survived |
| 25 | 2021 | Hunan, China | M | 45 | AIDS | Fatigue, diarrhea, anorexia | Not mentioned | Not mentioned | Multiple ulcers, bleeding | Ascending colon, transverse colon, descending colon, sigmoid colon | None | Colon  Biopsy | Amphotericin B | Survived |
| 26 | 2021 | Sichuan, China | M | 45 | AIDS, syphilis | Abdominal pain, diarrhea, fever, rash, anemia | Intra-abdominal, etroperitoneal, supraclavicular | 18 | Erosion | Ileocecum, entire colon | None | Colon, marrow, blood  Biopsy, culture | Fluconazol, sulfamethoxazole | Survived |
| 27 | 2021 | Hubei, China | F | 41 | AIDS | Abdominal pain, diarrhea, rash, fever, anemia | Neck and inguinal region | 36 | Ulcers | Colon, terminal ileum | None | Colon, marrow  Biopsy, culture | Itraconazole | Survived |
| 28 | 2022 | Zhejiang, China | M | 36 | AIDS, syphilis | Abdominal pain, diarrhea, oral ulcer, anemia | Intra-abdominal | 16 | Irregular marginal ulcer | Stomach, duodenum, ileocecum, ascending and transverse colon | Gastrointestinal bleeding | Stomach, duodenum, colon, blood  Biopsy, culture | Amphotericin B, Voriconazole, itraconazole | Survived |
| 29  (present) | 2022 | Hubei, China | M | 53 | AIDS | Abdominal pain, diarrhea, fever, weight loss, fatigue, anorexia, cough, anemia | Intra-abdominal, retroperitoneal, bilateral axillary and supraclavicular | 10 | Multiply scattered and swollen ulcers | Ileocecum, ascending colon, transverse colon, descending and sigmoid colon | None | Colon  Biopsy | Fluconazole | Survived |

Abbreviations: AIDS, acquired immunodeficiency syndrome; ITP, idiopathic thrombocytopenic purpura; PBC, primary biliary cirrhosis; TB, tuberculosis; STAT, signal transduction and activator of transcription
